# Neural repair mechanisms after ischemic stroke

**DOI:** 10.1186/s41232-025-00372-7

**Published:** 2025-03-17

**Authors:** Koshi Irisa, Takashi Shichita

**Affiliations:** Department of Neuroinflammation and Repair, Medical Research Laboratory, Institute of Science Tokyo, Bunkyo-Ku, Tokyo, 113-8510 Japan

**Keywords:** Ischemic brain injury, Neural repair, Inflammation, Reparative immune cells, Neural circuit reorganization

## Abstract

Ischemic stroke triggers inflammation that promotes neuronal injury, leading to disruption of neural circuits and exacerbated neurological deficits in patients. Immune cells contribute to not only the acute inflammatory responses but also the chronic neural repair. During the post-stroke recovery, reparative immune cells support the neural circuit reorganization that occurs around the infarct region to connect broad brain areas. This review highlights the time-dependent changes of neuro-immune interactions and reorganization of neural circuits after ischemic brain injury. Understanding the molecular mechanisms involving immune cells in acute inflammation, subsequent neural repair, and neuronal circuit reorganization that compensate for the lost brain function is indispensable to establish treatment strategies for stroke patients.

## Background

Stroke is the third leading cause of death worldwide and a major cause of severe disability over the long term [1]. Ischemic stroke, which accounts for approximately 80% of stroke cases, is caused by occlusion or stenosis of the cerebral artery [2]. Currently, treatment options for ischemic stroke are limited, with intravenous thrombolysis using recombinant tissue plasminogen activator (rt-PA) and thrombectomy being the primary approaches. However, these treatments can only be applied within a narrow time window after stroke onset, restricting their usage to about 5 to 10% of cases [3, 4]. In the long term, rehabilitation, which focuses on enhancing the spontaneous recovery of brain function through training to relearn skills lost after a stroke, remains the only available approach, as universally effective treatments for all stroke patients have yet to be developed.

Neurons in the ischemic core within the infarct region have lost their supply of oxygen and glucose, leading to calcium ion influx, reactive oxygen species (ROS) generation, and mitochondrial dysfunction, which ultimately result in necrotic neuronal death [5–7]. Necrosis of neurons activates the surrounding microglia, the resident immune cells in the brain, and disrupts the blood–brain barrier (BBB), leading to infiltration of neutrophils and macrophages several hours after a stroke [8]. T lymphocytes are activated by the cytokines and chemokines produced by infiltrating myeloid cells and contribute to post-stroke inflammation. Inflammation and BBB breakdown increase microvascular permeability, leading to brain tissue swelling [9]. This acute inflammation and edema cause further damage to neurons surrounding the infarct lesion (penumbra), contributing to poor patient outcomes. The acute inflammatory response resolves approximately 1 week after the ischemic stroke [10], followed by the long-term inflammation and neural recovery phase. After the acute inflammation phase, activated resident and infiltrating immune cells in the penumbral region shift from promoting inflammation to supporting neural repair processes, such as enhancing neuronal survival, axon sprouting, remyelination, and synaptogenesis. The recovery phase concludes within several months to a few years, leaving any remaining neurological deficits as lasting sequelae [11].

After ischemic injury, it has been reported that neural circuits undergo reorganization, contributing to the recovery or compensation of lost neurological function. Rehabilitation interventions within approximately 3 months after a stroke can help a patient partially regain mobility and daily life activities [12–15], and it has been reported that rehabilitative training promotes network remodeling, including distant regions both within the cortex and in subcerebral areas [16]. In addition to neuronal repair facilitated by reparative immune cells, activity-dependent neural circuit compensation is thought to occur. Advances in functional neuroimaging techniques—such as functional magnetic resonance imaging (fMRI) [17, 18], voltage-sensitive dye (VSD) imaging [19], and calcium imaging [20]—have enhanced our understanding of the dynamics of functional connectivity after a stroke. To improve the functional prognosis of stroke patients, two key considerations can be proposed as therapeutic targets: how to protect intact neural circuits and how to promote the remodeling of neural circuits to compensate for those that are lost. Treatments targeting immune cells or promoting neural circuit remodeling through axonal growth and neurogenesis are still in the early stages of development [21, 22]. Elucidating the mechanisms of time-dependent interactions between immune cells and neurons, as well as the processes of neural circuit reorganization after brain injury, will bring significant advances in treatment for stroke patients.

### Time-dependent roles of immune cells in the ischemic brain

#### Sterile inflammation induced by DAMPs after stroke

**Fig. 1:**
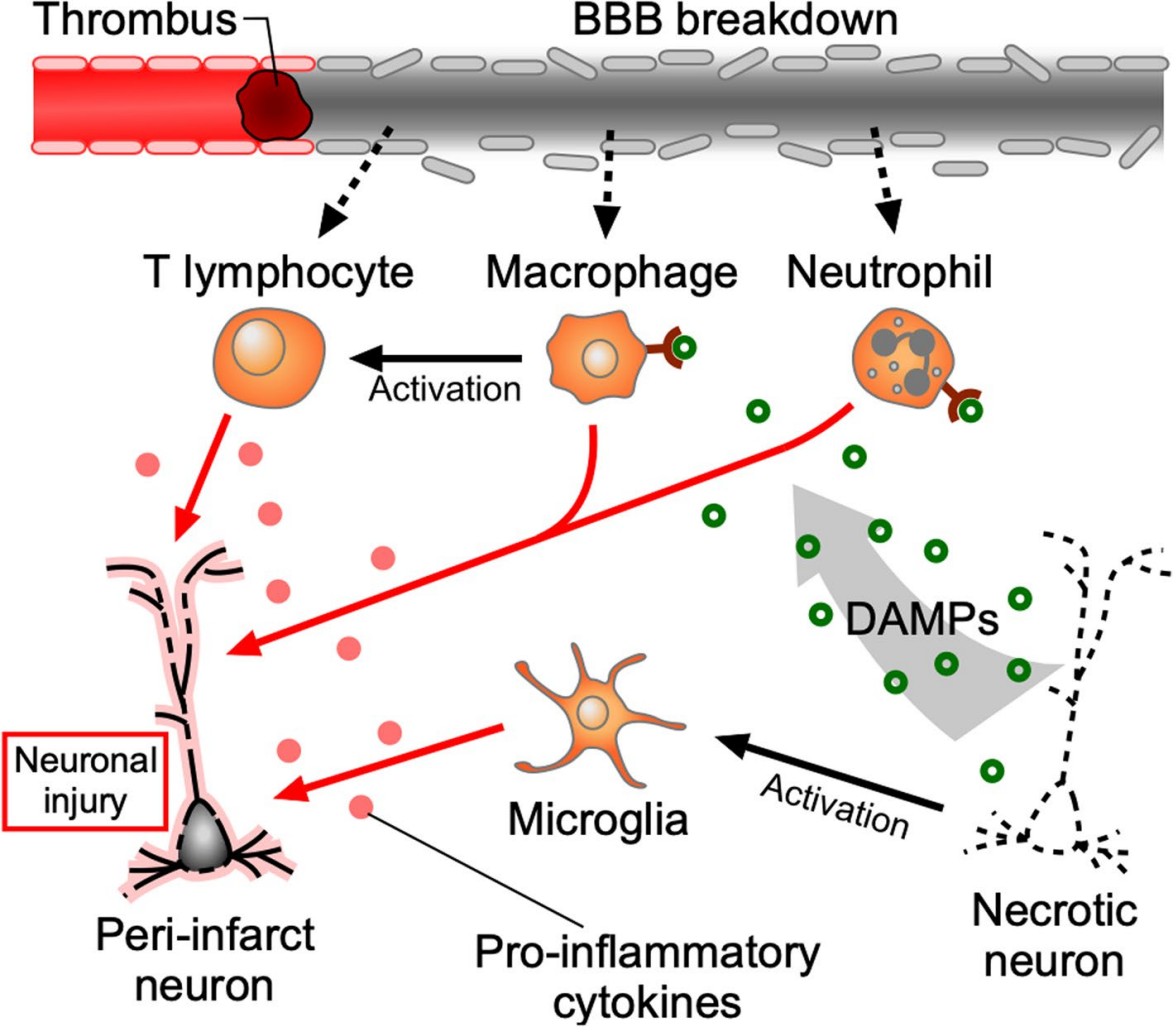
Sterile inflammation induced by DAMPs during acute inflammation phase. Focal vascular occlusion immediately induces necrotic neuronal death due to the lack of oxygen and glucose supply. Microglia, brain-resident myeloid cells, are activated in response to neuronal death and contribute to peri-infarct neuronal damage by producing cytokines such as TNFα and IL-1β, which exert direct neurotoxic effect or exacerbate cerebral inflammation and edema. Neutrophils and macrophages infiltrate through the disrupted BBB and are activated by DAMPs released from necrotic neurons to produce cytokines such as TNFα, IL-1β, and IL-6. T lymphocytes infiltrate following myeloid cells, are activated by cytokines released from myeloid cells, and produce IFNγ, IL-17, and IL-21, leading to harmful effects

In the focal ischemic area, necrotic cells release endogenous molecules, including damage-associated molecular patterns (DAMPs), such as high mobility group box 1 (HMGB1), peroxiredoxin family (PRXs), heat shock proteins, and adenosine triphosphate (ATP) [23–25]. These intracellular molecules activate brain-resident and infiltrating immune cells via pattern recognition receptors (PRRs). Toll-like receptors (TLRs), mainly TLR2 and TLR4, and the receptor for advanced glycation end-products (RAGE) are key receptors that recognize DAMPs derived from necrotic neurons and glial cells. Activated immune cells produce various inflammatory cytokines, contributing to secondary damage to surviving neurons in the peri-infarct area. Such post-stroke inflammatory responses, occurring in the absence of pathogens, are a typical example of sterile inflammation (Fig. 1).

#### Acute inflammatory response of each immune cell type

Microglia, the resident immune cells in the brain, are quickly activated in response to ischemic stroke [26]. Activated microglia produce cytokines, such as tumor necrosis factor-α (TNF-α) and interleukin-1β (IL-1β), which exert direct cytotoxic effects and phagocytose damaged neurons in the peri-infarct.

Neutrophils are the first immune cells to infiltrate through the disrupted BBB, within 6 h after a stroke [8, 27, 28], with their accumulation peaking within a few days [29–31]. Neutrophils exacerbate injury of peri-infarct cells by releasing inflammatory cytokines, such as TNF-α, IL-1β, and IL-6 [32, 33]. Extracellular traps released from activated neutrophils in the lesion site reduce neovascularization and increase BBB damage [34–36]. The infiltration of neutrophils requires the adhesion pair very-late-antigen-4 (VLA-4) and vascular cell adhesion molecule 1 (VCAM-1). The antibody-mediated depletion of infiltrating neutrophils improves neurological outcomes and reduces infarct volume in the acute inflammatory phase [8].

Monocytes and macrophages then infiltrate into ischemic regions following BBB breakdown, a major post-ischemic event, and further produce pro-inflammatory factors. Infiltrating macrophages are activated by recognizing inflammatogenic DAMPs via TLRs or RAGE [37]. Signaling through adapter molecules, such as MyD88 and TRIF, in the downstream cascade of TLRs activates pathways leading to the production of inflammatory cytokines via NF-κB and the induction of interferon pathways [25, 38].

T lymphocytes infiltrate the ischemic brain after myeloid cells and are activated by their released cytokines. Previous studies have reported that T lymphocytes cause harmful effects for acute inflammation by producing cytokines such as interferon-γ (IFNγ), IL-17, or IL-21, thereby exacerbating post-stroke inflammation and pathologies [39–42].

#### Reparative roles of immune cells following the acute inflammatory phase

**Fig. 2:**
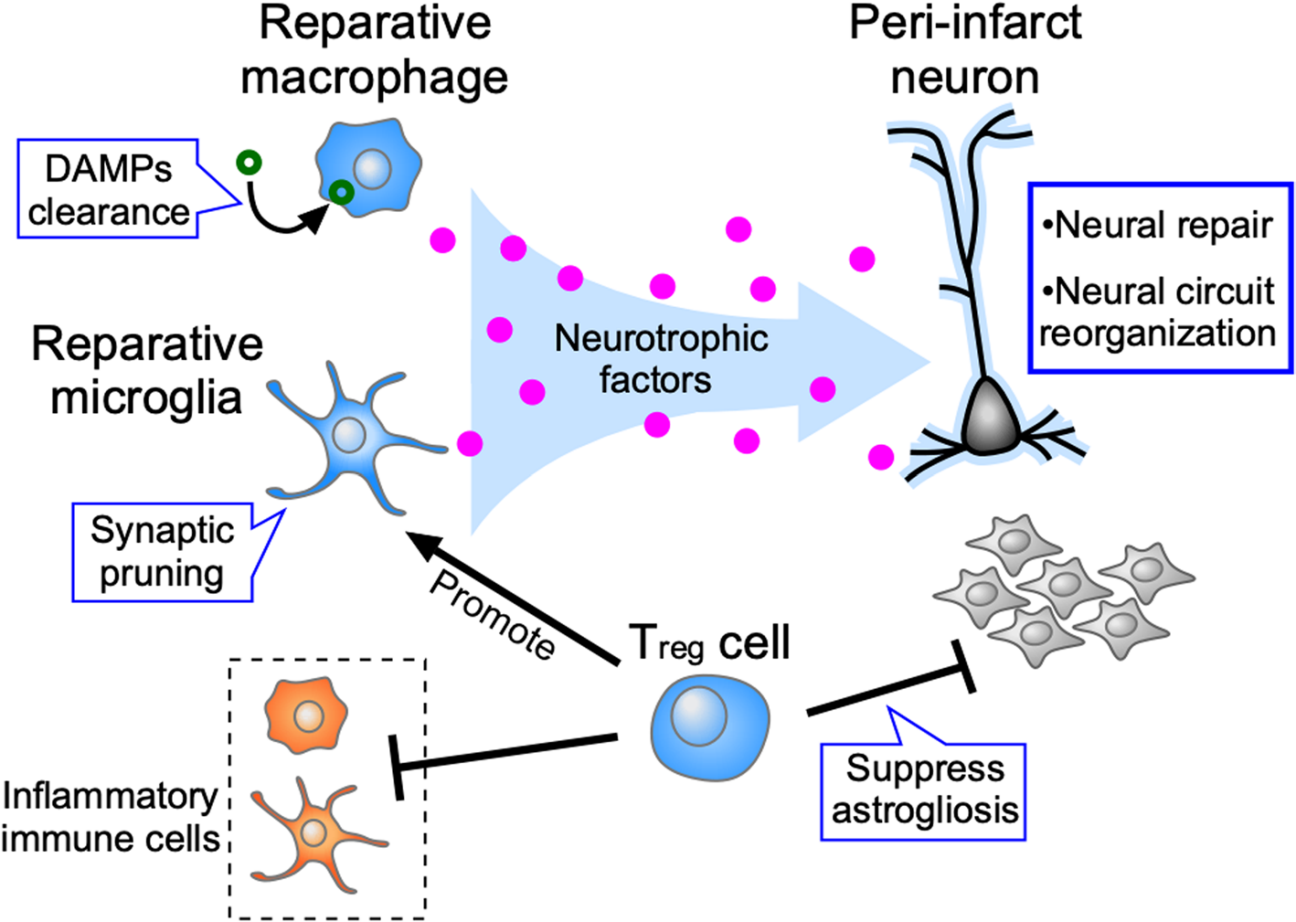
Reparative immune cell responses during recovery phase in the peri-infarct region. During the recovery phase, immune cells change from inflammatory to reparative functions. Reparative macrophages remove DAMPs via scavenger receptors such as MSR1 and MARCO, while microglia contribute to neural circuit reorganization by pruning unnecessary synapses. Both reparative macrophages and microglia produce neurotrophic factors, including OPN and IGF1, which support the protection and repair of damaged neurons in the peri-infarct region. T_reg_ cells that infiltrate 1–2 weeks after ischemic injury mitigate harmful inflammation caused by macrophages and microglia. T_reg_ cells also promote the activity of reparative microglia by producing OPN and suppress astrogliosis by producing amphiregulin

Some activated immune cells change their roles from acute inflammation to chronic neural repair during post-stroke recovery (Fig. 2). Microglia also change their functions from pro-inflammation by producing cytokines and chemokines to neural repair by producing neurotrophic factors [43]. Reparative microglia produce neurotrophic factors, including osteopontin (OPN), insulin-like growth factor 1 (IGF1), fibroblast growth factors (FGFs), hepatic growth factor, and growth differentiation factor 15 (GDF15), within a few days following a stroke. These factors play crucial roles in generating beneficial neuro-immune interactions for white matter repair [44–46]. It has also been reported that microglia are involved in the removal of unnecessary synapses after a stroke, contributing to increased synaptic turnover [47].

Macrophage infiltration peaks a few days after stroke onset and decreases within approximately 1 week [32, 33], during which the role of macrophages transitions from promoting inflammation to facilitating tissue repair between 3 and 6 days post-stroke [48, 49]. In the recovery phase, the scavenger receptors MSR1 and MARCO, expressed by reparative macrophages, play an important role in removing DAMPs from ischemic brain tissue [48]. Reparative macrophages also release neurotrophic factors, contributing to brain tissue reconstruction and neuronal repair [50].

Immunosuppressive regulatory T (T_reg_) cells, a subpopulation of T lymphocytes, infiltrate into the injury site within 1 to 2 weeks after ischemic stroke and accumulate for more than 1 month after stroke onset [51–54]. T_reg_ cells reduce harmful inflammation caused by microglia and macrophages by releasing various anti-inflammatory cytokines such as IL-10, and TGF-β [51, 53, 55, 56]. Amphiregulin, produced by T_reg_ cells, is a key molecule that suppresses astrogliosis and reduces neuronal damage [51]. T_reg_ cells also produce osteopontin, which enhances the activity of reparative microglia, promoting oligodendrogenesis and white matter repair [57].

### Reorganization of neural circuits

#### Morphological changes in neural circuits after brain injury

**Fig. 3:**
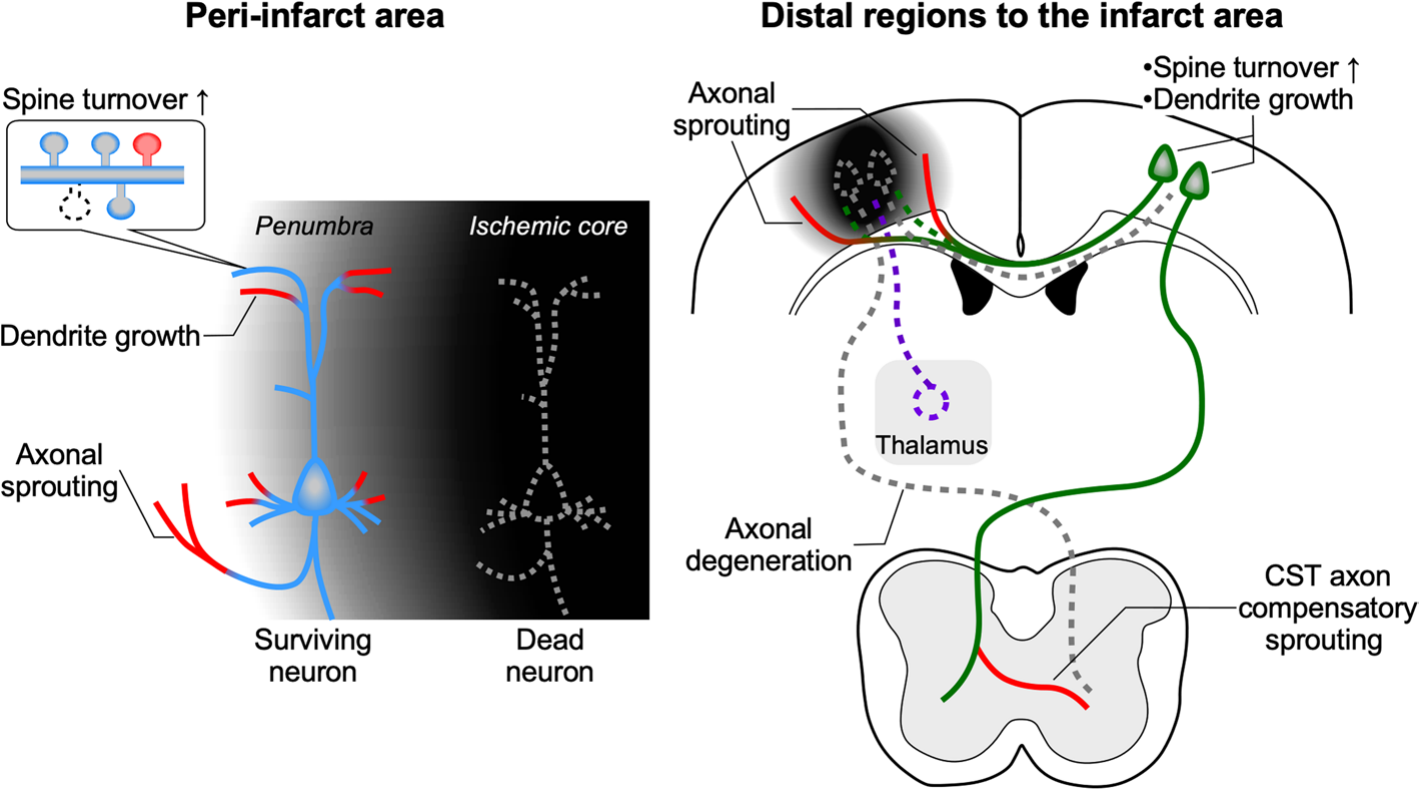
Morphological changes in neurons after ischemic brain injury. After an ischemic stroke, neural reorganization is observed as morphological changes in neurons, occurring not only in the peri-infarct region but also in the intact contralateral region and subcerebral areas. An increased rate of spine turnover is evident in both the penumbra and the contralateral regions during the first week following a stroke. In the subsequent weeks after ischemic injury, dendritic length also increases in the penumbra and contralateral side. Neurons projecting from the contralateral cortex to the ischemic core sprout axons into the peri-infarct region and survive, whereas thalamocortical neurons are lost. Axons of CST neurons in the undamaged hemisphere extend to the contralateral side at the spinal level, contributing to the compensation of motor impairments after a stroke

The cerebral cortex plays a crucial role in higher brain functions, such as motor control and sensory perception, by forming complex neural circuits through connections among neurons within functionally specialized cortical areas, as well as between cortical and subcortical neurons. The impact of neuronal death caused by focal ischemic stroke is not limited to the infarcted cerebral region; it also broadly affects neural circuits connected in this area. For instance, it has been reported that thalamocortical neurons projecting to the damaged cortical regions disappear within 48 h after a stroke [58]. After the acute phase, during which dysfunction of the neural circuit occurs, reorganization of the neural circuit is facilitated through synaptogenesis, dendritic growth, and axonal sprouting (Fig. 3).

After focal ischemia, reduction and elongation of dendritic spines are observed in the peri-infarct region within 24 h [59]. Subsequently, within 1 week, increased formation and elimination of spines, indicating an elevated turnover rate, occur in both the infarct site [19] and in the contralateral cerebral area [60]. In this period, axonal sprouting begins in the peri-infarct region [61] and remains robustly present even after 1 month [62]. The period when the spine turnover rate and axonal sprouting increase, indicating active reorganization of neural circuits, overlaps with the acute inflammation phase. Neurons projecting from the contralateral cortex to the ischemic core survive for 48 h after stroke [58]. Additionally, callosal projections to the injured site exhibit axonal sprouting in the perilesional area 4 weeks after thermocoagulation injury [63]. Descending axons from the undamaged motor cortex, called corticospinal tract (CST), sprout collaterals to the contralateral side, which has lost its neural connections [64, 65]. In non-human primates, ischemic injury to the primary motor cortex hand area increases reciprocal connections with the ventral premotor cortex and primary somatosensory cortex [66]. A localized ischemic stroke in a hemisphere alters even those connections between distant cortical areas and those long-projecting to the subcerebral regions, which may contribute to the compensation of functional neural circuits.

#### Remodeling of functional connectivity after brain injury

Neuroimaging techniques such as fMRI and VSD have been used to investigate cerebral functional connectivity after ischemic brain injury [67]. A study combining optogenetic stimulation using channelrhodopsin-2 (ChR2) and VSD demonstrated that cortical functional connectivity globally decreases 1 week after stroke onset but partially recovers by 8 weeks [68]. Focal ischemic injury to the primary somatosensory cortex (S1) induces redistribution of sensory responses, not only within the injured S1 [69] or contralateral intact S1 [60] but also extending to the motor cortex [70]. Intracortical microstimulation (ICMS) revealed that cortical motor output involves a reduced area for complex movements, whereas the area for simple movements expands [71]. fMRI is a non-invasive imaging technique that measures brain activity by detecting the temporal correlation of blood-oxygenation-level-dependent signals, making it suitable for use in humans. Many studies using fMRI in human stroke patients have revealed changes in functional connectivity between cortical regions [17]. Abnormal synchronization between cortical and subcortical structures, including the striatum, thalamus, and hippocampus, observed 2 weeks after a stroke gradually recovers over time [72]. Although reports on morphological and functional connectivity have demonstrated that neural networks undergo reorganization after stroke, it has been suggested that motor impairments may also involve newly developed abnormal interactions between cortical regions distant from the ischemic lesion. In the post-stroke brain, identifying functional and structural neural circuits is being explored as a means to develop novel therapeutic interventions [73, 74].

### Therapeutic interventions to promote neural circuit reorganization after stroke

#### Rehabilitative treatments

Rehabilitative intervention is widely implemented to improve motor dysfunction after a stroke. Patients who started rehabilitative training within a few months after a stroke showed improvements in motor function [12–15]. It has also been reported that rehabilitation within 6 months after a stroke is positively correlated with long-term mortality [75]. There are reports that initiating training within 24 h after ischemic brain injury may be harmful, but consensus indicates that starting rehabilitation within 2 weeks is highly effective [76]. Such therapeutic intervention is thought to assist in the reorganization of neural circuits during the recovery phase, and it has been demonstrated that they actually promote structural and functional changes in neural network.

The increasing dendritic arborization of layer 3 or layer 5 pyramidal cells in the motor cortex on the contralateral side of an ischemic lesion is enhanced both by rehabilitation [77, 78] and by housing in an enriched environment [79]. In spine turnover after a stroke, newly formed spines become more stable through rehabilitative training [80]. Forced limb use after intracerebral hemorrhage promotes axonal sprouting from the motor cortex to the red nucleus and facilitates the recovery of cortical motor function [81]. Constraint-induced movement therapy (CIMT) promotes an increase in the sprouting of CST neuron axons crossing to the contralateral side of the cervical spinal cord [64]. It also enhances the number of corticospinal projections originating from the peri-infarct motor cortex, contributing to functional recovery [82]. Chemogenetic stimulation specific to CST neurons, combined with rehabilitative training, enhances the midline-crossing sprouting of CST fibers, resulting in improved motor function recovery [83]. These results indicate that activity-dependent neural circuit reorganization is essential for functional recovery. In humans, advanced MRI techniques have demonstrated that rehabilitation positively influences the reorganization of structural and functional connection [84]. Despite the establishment of effective rehabilitative treatments, many stroke patients continue to experience long-term neurological deficits. In fact, several large intervention trials targeting motor recovery have shown improvements in participants’ motor performance; however, in most cases, the degree of improvement was trivial [85]. These findings suggest that rehabilitation aimed at promoting spontaneous circuit remodeling after brain injury has limitations in fully restoring brain function.

#### Therapeutic approaches for axonal regeneration and synaptic plasticity based on molecular mechanisms

Research investigating the molecular mechanisms underlying neural circuit remodeling is also progressing, and several neurotrophic factors critical for axonal regrowth have been identified. Transcriptome analysis of cortical neurons that project axons near the lesion site following ischemic injury revealed increased expression of growth factors, cell adhesion molecules, axon guidance molecules, and cytoskeletal modifiers in mice [62]. Brain-derived neurotrophic factor (BDNF) and insulin-like growth factor (IGF-1) are well-known neurotrophins. It has been reported that various neurotrophins are induced by exercise therapy [86, 87]. Expression levels of Lingo1 and BDNF, genes associated with plasticity and axonal sprouting, fluctuate based on timing after cerebral infarction and the target projection site [88], suggesting that axonal regeneration is mediated by neurotrophins in an activity-dependent manner. A treatment combining IGF-1 and OPN promotes CST fiber regeneration and CST-dependent functional restoration [89].

Axon regeneration in the central nervous system is limited, partly due to axon growth inhibitors such as Nogo-A, myelin-associated glycoprotein, oligodendrocyte-myelin glycoprotein, chondroitin sulfate proteoglycans, RhoA, and semaphorin 3A, whose expression is elevated around the injury site [64, 90]. Therapies aimed at promoting neural circuit reorganization by suppressing these axon growth inhibitors are being developed. Injecting chondroitinase ABC or anti-Nogo-A antibodies into the spinal cord increases the number of sprouting axons from the intact cerebral cortex to the spinal cord in rats after stroke [91–93]. Additionally, injecting chondroitinase ABC into the penumbral region surrounding the infarct enhances synaptic density and facilitates motor recovery [94]. The administration of a semaphorin 3A inhibitor to the peri-infarct area promotes axonal growth and improves neurological deficits after ischemic brain injury [95]. However, clinical trials of myelin-associated glycoprotein, neurite outgrowth inhibitor proteins, and chondroitin sulfate proteoglycans have shown limited therapeutic efficacy [96]. Given that functional recovery requires the establishment of appropriate neural circuits, combining treatments that enhance neuronal plasticity with stimulation therapies, such as transcranial magnetic stimulation, and rehabilitative interventions may be necessary. Among such approaches, edonerpic maleate has been shown to promote neuronal plasticity through facilitating experience-driven synaptic glutamate AMPA receptor delivery, thereby accelerating motor function recovery after cortical injury in rodents and non-human primates [97].

## Conclusions

Elucidating the mechanisms of neural circuit destruction caused by immune inflammation following cerebral infarction and the subsequent activity-dependent reorganization of circuits, which involves reparative immune cells, is crucial for developing treatments for stroke patients. Although substantial progress has been made in understanding the biphasic neuro-immune interaction, effective therapeutic approaches for ischemic stroke have yet to be established. In the future, integrating the microscopic understanding of molecular mechanisms involved in neural repair—including neurons, immune cells, and glial cells—with the macroscopic perspective of functional connectivity remodeling will be a critical challenge for advancing approaches to neural recovery after ischemic stroke.

## Data Availability

Not applicable.

## Abbreviations

ATP: Adenosine triphosphate
BBB: Blood-brain barrier
BDNF: Brain-derived neurotrophic factor
CIMT: Constraint-induced movement therapy
CST: Corticospinal tract
ChR2: Channelrhodopsin-2
DAMPs: Damage-associated molecular patterns
FGFs: Fibroblast growth factors
GDF15: Growth differentiation factor 15
HMGB1: High mobility group box 1
ICMS: Intracortical microstimulation
IFNγ: Interferon-γ
IGF1: Insulin-like growth factor 1
IL: Interleukin
MARCO: Macrophage receptor with collagenous structure
MSR1: Macrophage scavenger receptor 1
OPN: Osteopontin
PRR: Pattern recognition receptor
PRXs: Peroxiredoxin family
RAGE: Advanced glycation end-products
ROS: Reactive oxygen species
S1: Primary somatosensory cortex
TLRs: Toll-like receptors
TMS: Transcranial magnetic stimulation
TNF-α: Tumor necrosis factor-α
T_reg_: Regulatory T
VCAM-1: Vascular cell adhesion molecule 1
VLA-4: Very-late-antigen-4
VSD: Voltage-sensitive dye
fMRI: Functional magnetic resonance imaging
rt-PA: Recombinant tissue plasminogen activator
